# Physical activity in patients with rheumatoid arthritis - an agile lifelong behaviour: a qualitative meta-synthesis

**DOI:** 10.1136/rmdopen-2021-001635

**Published:** 2021-05-07

**Authors:** Emma Swärdh, Christina Opava, Nina Brodin

**Affiliations:** 1 Department of Neurobiology, Care Sciences and Society, Division of Physiotherapy, Karolinska Institutet, Huddinge, Sweden; 2 Department of Orthopaedics, Division of Physiotherapy, Danderyds Sjukhus AB, Stockholm, Sweden

**Keywords:** qualitative research, rehabilitation, arthritis, rheumatoid

## Abstract

**Background:**

Physical activity (PA) in rheumatoid arthritis (RA) is considered a cornerstone in the treatment. To highlight aspects involved in supporting a positive PA behaviour, it is important to understand the patients’ perceptions of the phenomenon.

**Objective:**

The aim of this qualitative meta-synthesis was to explore and synthesise patient perceptions of PA in RA.

**Methods:**

A purposeful search was conducted across three online databases (PubMed, CINAHL and Web of Science). The methodological quality of the included studies was appraised, and data were extracted and analysed using an interpretive inductive thematic synthesis.

**Results:**

Fifteen studies met the inclusion criteria and were included. PA was identified as an agile lifelong behaviour, with one main theme: The disease as a persistent catalyst for or against PA illustrating how the constant presence of the disease itself underlies the entire process of a life with or without regular PA. Seven subthemes: ‘considering aggravated symptoms’, ‘acknowledging the impact on health’, ‘becoming empowered and taking action’, ‘keeping informed to increase awareness’, ‘creating body awareness’, ‘dealing with social support’ and ‘feeling satisfied with circumstances and achievements’ were interpreted as facilitators and/or challenges.

**Conclusion:**

This synthesis has identified PA as an agile lifelong behaviour in which the disease pervades all aspects of an individuals’ perception of PA. Placed in a theoretical context, our findings outline a model for tailoring PA support to the drivers and determinants of a certain individual, which will improve clinical practice for the benefit of both health professionals and patients with RA.

**Key messages**

**What is already known about this subject?**

- Physical activity (PA) in rheumatoid arthritis (RA) is considered a cornerstone in the treatment of RA and patients’ perceptions of phenomena related to PA have been studied over two decades in order to gain understanding of this complex behaviour.

**What does this study add?**

- This qualitative synthesis of 15 studies identifies PA as an agile lifelong behaviour with a main theme illustrating how the constant presence of the disease itself underlies the entire process of balancing facilitators and challenges to regular PA.

**How might this impact on clinical practice or further developments?**

- Placing the perceived facilitators and challenges to PA in a theoretical context, our findings outline a model for tailoring PA support to the drivers and determinants of a certain individual, which will be helpful to both health professionals and patients with RA.

## Introduction

Good-quality evidence has accumulated over the past two decades on the effectiveness of aerobic and muscle-strengthening physical activity (PA) to reduce disease-related symptoms and comorbidity risk in people with rheumatoid arthritis (RA).^1–3^ Since PA is effective and safe, it is recommended to be included in the standard care of patients with RA.^4^ Nevertheless, PA levels remain low in people with RA^5–7^ and a number of factors challenge the participation in and maintenance of regular PA. Such factors include, for example, time and cost, pain, fatigue and activity limitation, poor self-regulation skills and low autonomous motivation, as well as a lack of knowledge and reluctance on the part of health professionals (HPs) to implement evidence-based PA guidelines.^8–11^

PA is a complex behaviour. Moving from a sedentary state to a more physically active state can be challenging for an individual, who may benefit from facilitating factors but also needs to manage several potential barriers. Facilitating factors and barriers for behaviour change include, but are not limited to, current and past behaviour, beliefs, emotions and context, social aspects and physical abilities.^12 13^ Thus, HPs who aim to support improved PA behaviour among their patients should have a good understanding of these aspects, as well as the complex interplay between them. Findings from studies with qualitative design may be useful for understanding the nature of facilitators in reinforcing PA promotion and the barriers that need addressing.

In recent decades, a growing body of knowledge on the experience and perception of PA in people with RA has emerged from studies with qualitative designs.^14–17^ A synthesis of their findings would identify the knowledge gaps that need to be addressed in future research and would also help HPs to better grasp their essence and make use of them. A previous meta-synthesis focused solely on the experience of exercise participation in people with RA.^18^ However, there is currently a need for a synthesis that has a broader scope and includes a wide variety of phenomena related to PA in people with RA. Thus, the aim of our study was to review studies with a qualitative design and synthesise their findings in order to achieve a more comprehensive understanding of phenomena related to perceptions of PA in RA.

## Method

This study used an interpretivist approach^19^ for conducting a qualitative meta-synthesis^20^ on phenomena related to PA. A meta-synthesis connects and synthesises findings from several individual qualitative studies at a higher abstraction level aiming at understanding the perceptions of different individuals from different contexts and make the findings more accessible for clinical practice.^21^ The review followed the Enhancing Transparency of Reporting the Synthesis of Qualitative research statement^22^ in order to report the different stages of synthesis and methods for qualitative findings (online supplemental file 1).

10.1136/rmdopen-2021-001635.supp1 Supplementary data

### Search strategy and inclusion criteria

**Figure 1:**
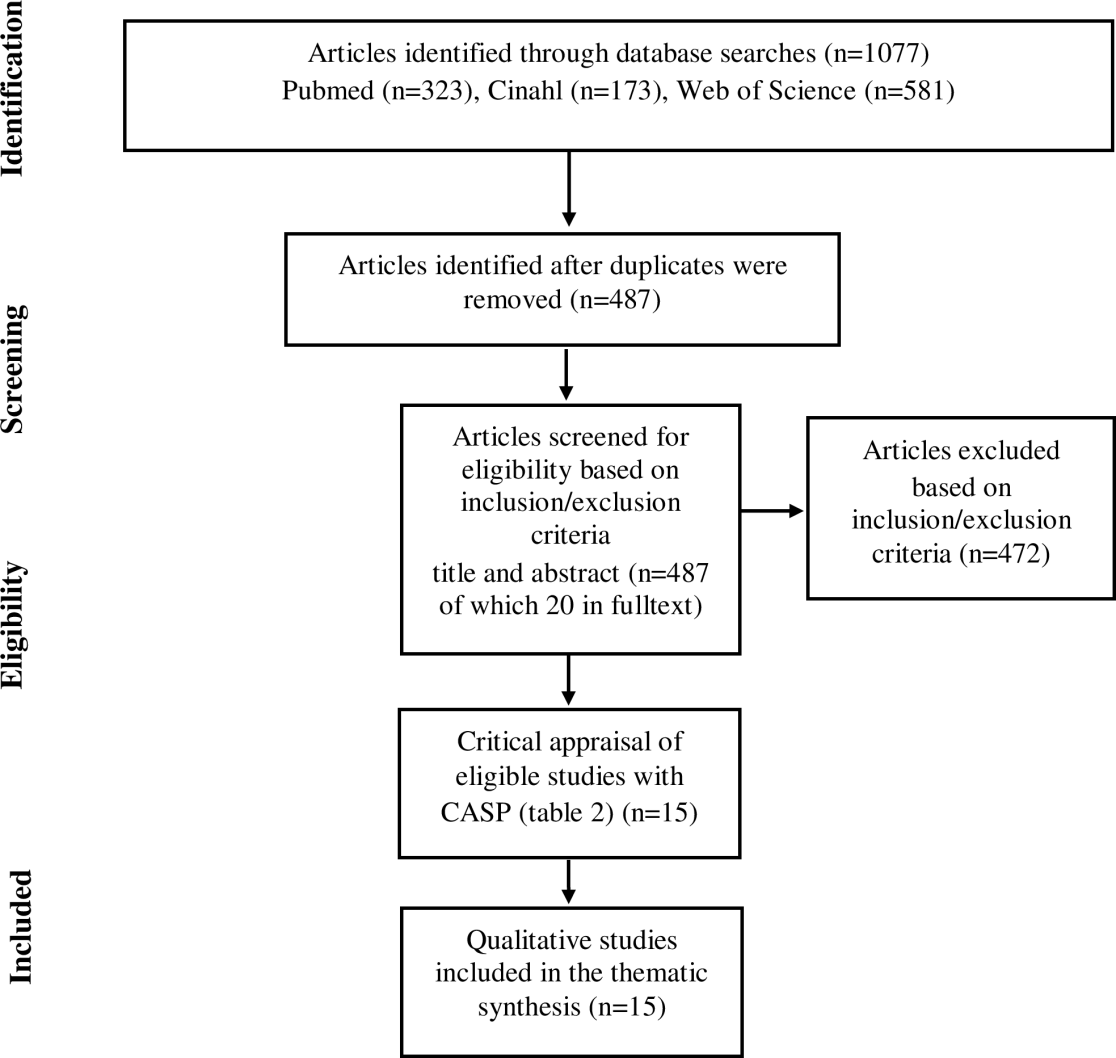
The different phases of the search process. CASP, Critical Appraisal Skills Programme.

A purposeful sampling strategy^23^ was applied and performed through a search of electronic databases (PubMed, CINAHL and Web of Science) using combinations of key search terms: ‘qualitative methods, qualitative study, interviews, focus groups, PA, exercise and RA’ (online supplemental file 2). To ensure maximum retrieval, no date limitation was used on the search which was performed up to 31 December 2020. A modified Preferred Reporting Items for Systematic Reviews and Meta-Analyses flow chart^24^ was used to illustrate the purposeful search and selection process (figure 1). Studies using a qualitative research design were included if they explored perceptions of different phenomena related to PA in individuals with RA, were empirical, published in English in peer-reviewed journals and included only informants with RA who were at least 18 years of age. Studies were excluded if they were published as short reports or letters, used questionnaires to collect data, explicitly explored body-mind exercises such as yoga, mindfulness or tai chi or PA interventions supported by e-Health or m-Health technology. Two independent reviewers (ES and NB) screened the titles and abstracts, excluded studies that did not meet the inclusion criteria and evaluated full-text versions for eligibility. Further, backtracking of the reference lists of the retrieved articles was performed.

10.1136/rmdopen-2021-001635.supp2 Supplementary data

#### Quality appraisal/quality assessment method

The quality of the studies included in the synthesis was screened and appraised using the 10-item Critical Appraisal Skills Programme (CASP) checklist for qualitative research.^25^ Two researchers (NB and ES) independently appraised all of the included studies and resolved any discrepancies through discussion (table 1). An independent researcher (Adrienne Levy-Berg), familiar with qualitative methods, appraised the quality of the included studies published by the authors of the present study.

**Table 1:** Characteristics of quality appraisal using the CASP quality tool for qualitative research

	Was there a clear statement of aims of the research?	Is a qualitative methodology appropriate?	Was the research design appropriate to address the aims of the research?	Was the recruitment strategy appropriate to the aims of the research?	Were the data collected in a way that addressed the research issue?	Has the relationship between the researcher and participants been adequately considered?	Have ethical issues been taken into consideration?	Were the data analysis sufficiently rigorous?	Is there a clear statement of findings?	How valuable is the research?
Baxter *et al*^28^	x	x	–	–	x	x	x	x	x	x
Bearne *et al*^27^	x	x	–	x	x	x	x	–	x	x
Brodin *et al*^14^	x	x	x	x	x	x	x	x	x	x
Crowley and Kennedy^29^	x	x	x	–	x	–	x	–	x	x
Eurenius *et al*^37^	x	x	x	x	x	–	x	x	x	x
Kamwendo *et al*^15^	x	x	x	–	x	–	–	x	x	x
Lange *et al*^30^	x	x	x	x	x	x	x	x	x	x
Larkin *et al*^16^	x	x	x	–	x	x	x	x	x	x
Law *et al*^31^	x	x	x	x	x	x	x	x	x	x
Loeppenthin *et al*^34^	x	x	x	x	–	x	x	x	x	x
Nichols *et al*^32^	x	x	x	x	x	x	x	x	x	x
Swärdh *et al*^17^	x	x	x	x	x	x	x	x	x	x
Swärdh *et al*^33^	x	x	x	x	x	x	x	x	x	x
Thomas *et al*^35^	x	x	x	x	x	–	x	–	x	x
Withall *et al*^36^	x	x	x	–	x	x	x	x	x	x

x, present in study, -, absent from the study.

CASP, critical appraisal skills programme.

### Data extraction

All of the included studies were read independently by two researchers (ES and NB) and all text related to the findings and quotes from each study were extracted for analysis, as well as the findings in abstracts and conclusions. The following data were also extracted in order to provide a summary of each of the included studies: author, year, country, research aim, sampling methods, participant characteristics (number, gender, age, disease duration) and method of data collection and analysis.

### Data synthesis and analysis

An interpretive inductive thematic synthesis, in accordance with the techniques of Thomas and Harden,^26^ was conducted by two of the authors (ES and NB) and discussed with the third author who was a peer expert (CO). Thematic synthesis involves identifying key concepts across studies based on the findings from each study and creating themes that go beyond the content of the original studies. The analysis comprised three stages: (1) line-by-line coding of the findings of the studies, (2) developing descriptive themes and 3) generating a higher level of analytical themes.^26^ The studies were first read several times to familiarise with all the text to be analysed from each study and codes were identified by one of the authors (ES) and grouped together based on their similarities and differences. The codes were organised to form descriptive themes by two of the authors (ES and NB), who subsequently developed analytical themes inductively (theme and subthemes) by interpreting the descriptive themes and answer the aim of the present meta-synthesis. Data were repeatedly explored, compared and contrasted across the studies and the main theme and subthemes were discussed and reviewed with CO in order to generate new conceptual interrelationships and understandings. A thematic map that interpreted the interrelationship between the main theme and the subthemes was developed as a final step.

## Findings

### Literature search results

Fifteen primary studies with qualitative designs including a total of 233 individuals with RA (177 women, 56 men, aged 21–87 years) were included in the review and synthesis^14–17 27–37^ (table 2). The studies were conducted in Denmark, Ireland, New Zealand, Sweden and the UK. All studies captured patient perspectives on PA but varied in terminology, with eight studies using the term ‘PA’^14–16 33–37^ and seven using ‘exercise’.^17 27–32^ The focus of the studies varied between perceptions of specific PA programmes, future PA programme designs, barriers and facilitators, maintenance, attitudes, effects, intensity, as well as PA in everyday life. Most studies included participants with low to high levels of PA, although PA levels were unclear^31^ or not specified in six studies.^15 16 28 29 36^ Most studies used purposive sampling but two used consecutive sampling^15 30^ and another used a snowball sampling method,^29^ while the sampling strategies were unclear in two of the studies.^16 28^ Sample sizes varied from eight to 19 participants. Most studies recruited their informants from rheumatology clinics, physiotherapy clinics or arthritis associations. Twelve studies used individual interviews,^14–17 27 28 30 32–35 37^ and three employed focus group methodology.^29 31 36^ Six studies used thematic analysis,^16 27–29 35 36^ four used phenomenographic analysis,^14 15 17 37^ three used content analysis^30 31 33^ and individual studies used systematic text condensation,^34^ and interpretative phenomenological analysis.^32^

**Table 2:** Characteristics of the included studies

Authors, year, Country	Research aim	Sampling methods	Participant characteristics	Data collection	Method of analysis
Baxter *et al,*^28^ 2016, New Zealand	Explore the perceived barriers, facilitators and attitudes to exercise in people with RA	Unclear sampling strategy Rheumatology outpatient clinic at a public hospital	n=8, (F=8, M=0) Age: m=62 years (56–82) Disease duration: m=6 years (5-29)	Semistructured individual telephone interviews	Thematic analysis
Bearne *et al,*^27^ 2017, United Kingdom	Explore participants’ experiences of EXTRA and consider refinements to EXTRA	Purposive sampling Attendance at the EXTRA sessions	n=12, (F=10, M=2) Age: m=57.8 years (32–87) Disease duration: m=33 months (12–65 months)	Semistructured individual interviews	Thematic analysis
Brodin *et al,*^14^ 2009, Sweden	Describe variation in the ways that individuals with RA understand how to determine the intensity of PA	Purposive sampling Physiotherapy clinics at hospitals	n=19, (F=12, M=7) Age: m=58.5 years (21–82) Disease duration: m=15 years (2–55)	Semistructured individual interviews	Phenomenographic analysis
Crowley and Kennedy,^29^ 2009, Ireland	Identify barriers and facilitators to exercise in RA and methods to increase compliance	Snowball sampling Arthritis Ireland Limerick branch meeting	n=12, (F=12, M=0) Age: 62.5 years (43–80) Disease duration: 15.6 years (1-33)	Focus group interviews	Thematic content analysis
Eurenius *et al,*^37^ 2003, Sweden	Describe variations in attitudes to PA in a group of people with RA	Purposive sampling Outpatient rheumatology unit at a hospital	n=16, (F=12, M=4) Age: Md=61.5 years (32–78) Disease duration: Md=16.5 years (1–45)	Semistructured individual interviews	Phenomenographic analysis
Kamwendo *et al,*^15^ 1999, Sweden	Achieve a better understanding of how patients with RA perceive and relate to PA in their everyday life	Consecutive sampling Outpatient RA clinic	n=10, (F=6, M=4) Age: m=56.5 years (42–68) Disease duration: m=10.9 years (1-28)	Individual interviews	Phenomenographic analysis
Lange *et al,*^30^ 2019, Sweden	Explore aspects of participation in moderate-intensity to- high-intensity exercise with person-centred guidance influencing the transition to independent exercise for older adults with RA.	Consecutive sampling The intervention arm of a randomised controlled trial evaluating moderate- to- high-intensity exercise intervention with person-centred guidance	n=16, (F=11, M=5) Age: M=70.6 years (67-76) Disease duration: m=14 years (3-45)	Semi-structured, individual, in-depth interviews	Qualitative content analysis
Larkin *et al,*^16^ 2017, Ireland	Explore the views of people who have RA on the design of a future PA intervention	Unclear sampling strategy Outpatient rheumatology clinic in an urban hospital	n=17, (F=12, M=5) Age: m=59.8 years (35–83) Disease duration: m=13.7 years (1-47)	Semi-structured individual telephone interviews	Inductive thematic analysis
Law *et al,*^31^ 2010, United Kingdom	Describe the perceptions of the effects of exercise on joint health among RA patients	Purposive sampling Department of Rheumatology, University health board	n=18, (F=12, M=6) Age: 59.1 years (23–76) Disease duration: (2.5 months–33 years)	Focus group interviews	Systematic content analysis
Loeppenthin *et al,*^34^ 2014, Denmark	Describe the meaning of PA maintenance	Purposive sampling The Danish Rheumatism Association and outpatient clinic of rheumatology	n=16, (F=12, M=4) Age: m=50 years (37–67) Disease duration: m=21 years (4–46)	Semi-structured individual interviews	Systematic text condensation
Nichols *et al,*^32^ 2017, United Kingdom	Explore participants’ experiences of the SARAH exercise trial and how successfully they adhered to the programme over time	Purposive sampling Four National health service trusts	n=14, (F=9, M=5) Age: M=61.4 years (44–82) Disease duration: 13.2 years (1-36)	Semistructured individual interviews	Interpretive phenomenological analysis
Swärdh *et al,*^17^ 2008, Sweden	Explore and describe ways of understanding exercise maintenance	Purposive sampling Four hospitals or primary healthcare physical therapy clinics	n=18, (F=14, M=4) Age: m=60 years (34–83) Disease duration: (3–53 years)	Semi-structured individual interviews	Phenomenographic analysis
Swärdh *et al,*^33^ 2020, Sweden	Explore perceptions of maintaining PA according to public health recommendations during the second year of an outsourced support programme.	Purposive sampling The outsourced 2 year PA support programme, and previously participation in semi-structured interviews after the first year of the programme.	n=18, (F=15, M=3) Age: m=66.5 years (48–72) Disease duration: Md=5 (1–25 years)	Semi-structured individual interviews	Conventional content analysis
Thomas *et al,*^35^ 2019, United Kingdom	Explore the experiences, perspectives and strategies employed by people with RA who are successfully engaging with regular PA	Purposive sampling Outpatient rheumatology clinic	n=15 (F12, M=3) Age: m=56 years (29–80) Disease duration: m=13 years (10 months–46 years)	Semi-structured individual interviews	Inductive thematic analysis
Withall *et al,*^36^ 2016, United Kingdom	Explore the views of people with RA regarding the feasibility and acceptability of potential PA programmes	Purposive sampling Rheumatology clinics at university and national hospitals	n=19, (F=15, M=4) Age: m=59.9 years (31–73) Disease duration: m=44.3 months (1–120)	Focus groups	Inductive thematic analysis

PA, physical activity; RA, rheumatoid arthritis; SARAH, The Strengthening And stretching for Rheumatoid Arthritis of the Hand.

### Quality appraisal

The methodological strength of the included studies varied (table 1). Thus, 13 studies adequately justified their research design, while two did not.^27 28^ Five studies did not adequately describe their recruitment strategies.^15 16 28 29 36^ Fourteen studies met the quality criteria for data collection but one study did not discuss data saturation or provide a justification for the sample size.^34^ Eleven studies discussed the relationship between the researcher and the participants; the other four studies^15 29 35 37^ failed to discuss their own role and potential bias and how this may have influenced the research process and findings of the study. One study did not confirm whether ethical approval had been sought.^15^ Thirteen studies provided adequate in-depth descriptions of the analysis process, the other two did not.^27 29^ Bias was addressed in all studies using one or more independent researchers in parts of the analysis. One study did not provide thick interpretative descriptions to support its findings.^35^ All studies used quotes to support their findings. All findings were discussed in relation to the original research aims and the credibility of the findings was discussed in all of the studies. All studies were appraised as valuable. Thus, none of the studies were excluded because of methodological flaws.

### Synthesis

**Figure 2:**
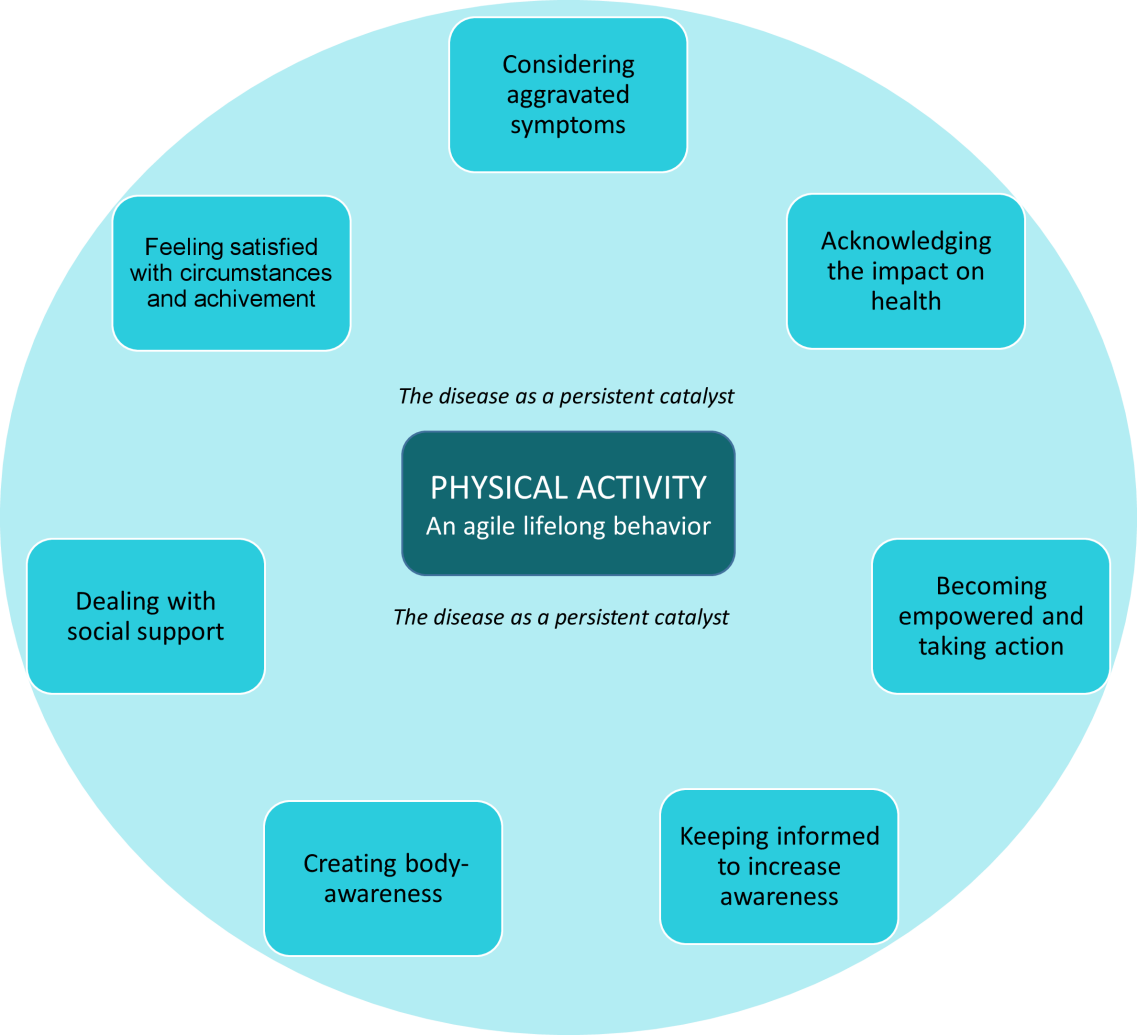
The thematic map describing the synthesis of PA perceptions in RA. PA, physical activity; RA, rheumatoid arthritis.

Across the studies and based on the thematic synthesis, we identified PA as an agile lifelong behaviour, with one main theme: The disease as a persistent catalyst for or against PA illustrating how the constant presence of the disease itself underlies the entire process of a life with or without regular PA (figure 2). The disease can act as a driving force to motivate PA by not letting the disease take over, but can also be perceived as a burden responsible for a person’s inability to be physically active. The disease also affects PA both positively and negatively by prompting different facilitators and/or challenges. Seven subthemes: *‘*considering aggravated symptoms’, ‘acknowledging the impact on health’, ‘becoming empowered and taking action’, ‘keeping informed to increase awareness’, ‘creating body awareness’, ‘dealing with social support’ and ‘feeling satisfied with circumstances and achievements*’* were interpreted as these facilitators and/or challenges for lifelong PA behaviour.

### Considering aggravated symptoms

This theme refers to individuals’ fears and concerns about aggravating the disease or its symptoms. Thus, concerns about the safety of PA may represent a challenge to performance and speculation as to whether PA helps or damages joints and/or worsens disease activity is common. Such concerns are present at the time of disease onset, as well as during flares or unpredictable events in more advanced disease stages. Other safety issues relate to fear of falling. Questions about whether all PA is suitable for individuals with RA or whether it should be adapted in order to not exacerbate the disease result in hesitation. Disease symptoms such as fatigue, pain, stiffness and decreased mobility can be determinants for disengaging in PA or considered as facilitators by representing minor obstacles towards a life with regular PA.

At first I was a bit apprehensive of what was going to happen…, you know, with these exercises…would (the exercises) be beneficial to me or not?(Bearne *et al*^27^)

you know it’s hard doing certain things for exercise but is that coz its exercise or is that because I shouldn’t be doing it? (Baxter *et al*^28^)

a barrier more when I’m walking…you’re constantly watching…especially when you have had a few bad falls… (Crowley *et al*^29^)

If you do something and it’s that painful, it must be doing your joints some damage (Law *et al*^31^)

### Acknowledging the impact on health

This theme illustrates the need to experience and perceive the physical and mental benefits of PA. Fear of losing independence or exacerbation of symptoms due to inactivity increases motivation for PA because of its well-known general benefits. Habitual and regular PA can act as a way of coping with the disease, keeping a strong body, decreasing symptoms and retaining independence. Insights, either self-experienced or gained through conversations with others, into the potential health benefits of regular PA can help individuals achieve a more active and conscious involvement in PA.

The joints are lubricated when I move, it doesn’t make me stiff but simply more agile, it gives me a better general condition and all that (Kamwendo *et al*^15^)

it becomes easier to get up from a chair, to use the toilet, so many things in your everyday life becomes easier (Loppenthin *et al*^34^)

I think it’d have a good effect…a lot. But finding something physical that you can manage and be able to start a bit gentle and work up. I think that would be good for…yes, my body and temper and everything. And seeing that you manage a bit more and more, I think that’d be very useful…you have to find something and get started (Eurenius *et al*^37^)

I feel exercise is necessary, essential and helpful for joint health (Law *et al*^31^)

(Without physical activity) I won’t be able to live anywhere near as normal life as possible which is what’s important to me (Thomas *et al*^35^)

What I’m thinking is, that I understand, that it’s vital. I need to move. And I, I mean, am I, if I want to have a good life, I need to continue exercising (Swärdh *et al*^33^)

### Becoming empowered and taking action

This theme encompasses individuals’ foci, goals, priorities and responsibilities related to PA. Taking an active role in creating healthy PA behaviour requires an individual to identify their own willpower and persistence to fight the disease, as well as self-discipline in which lame excuses for not engaging in PA are set to one side. Lapses and relapses in PA are common for individuals with RA and represent a real challenge. It is hard to resume PA routines and lack of motivation can be part of the problem. However, a plan and set goals, diaries, monitoring, focus and conscious prioritisation can get the ‘PA job’ done. Pushing yourself and identifying solutions for any problems help this process, although restarting and finding new motivation is another helpful strategy.

I have to grab myself every time - and say, well there is only yourself, so there is no excuse to stay home, then I just go—it’s my life, and I have to take responsibility for it (Loppenthin *et al*^34^)

…your goal…you write that in, and you work towards that…and it’s a reminder, it’s there, so you can turn back to the page and look at it…and, yeah, keep going (Bearne *et al*^27^)

Your mind is the most important thing because if your mind is right your mind will make you do exercise (Crowley *et al*^29^)

Introducing it into the routine and being able to manage time was probably the key difficulty…this is an activity that is now inbred within your general activity of a day and it becoming a habit is then just something that now evolves through naturally, and I think that’s a key difference for me (Nichols *et al*^32^)

You have to schedule it, and you have to stick to the time. You have to do it just when you’ve planned to and not put it off at all (Swärdh *et al*^17^)

The continuity, the scheduling, that you follow a programme. That’s what it’s about. My whole life, I’ve always been a planner, at work and all that, and if you don’t have any goals and no plan, then nothing happen. But here you could see you could have a plan, and it was easy to follow so there was nothing to it, reaching your goals worked fine *(*Swärdh *et al*^33^)

### Keeping informed to increase awareness

This theme encompasses awareness of different aspects of PA. Individuals want to learn, know and hear about why people with RA should be physically active, not just generally, but in relation to their disease. Questions related to recommendations for PA in people with RA such as optimal type of PA, frequency, duration and intensity are common. Also, insecurity and problems finding a balance between rest and activities suggest that there is a need for an individual approach to ‘the best type of PA for me’ by adapting the PA. This information can be provided by HPs through written material, face-to face meetings, smartphone apps or websites. No matter how, where or when the information is given, it is important that it is updated and coherent. However, HPs are not always willing to discuss and individual support are therefore not always available. In this respect, HPs should be experts and sounding boards when it comes to detailed and updated information about PA.

I knew what I could do and what I couldn’t do. Now I just have no idea. I mean I have asked the doctors and they say just do as much as you can (Baxter *et al*^28^)

we would love to have someone to go to who would tell you exactly what to do and what you could do and what you’re not supposed to do (Crowley *et al*^29^)

well someone that’s professionally trained I presume, for safety…They’d have to know about both RA and physical activity (Larkin *et al*^16^)

I would really like to know what they call exercise and whether or not it conforms to what I think is exercise (Law *et al*^31^)

### Creating body awareness

This theme embraces the ability to listen to the body and adapt PA to changes in physical status. A vital process in maintaining PA is thus learning to know your own body in relation to the disease, as well as recognising how different types and amounts of PA affect the body in different ways. Normal physical responses to gradually increased PA intensity confer a sense of having a ‘normal’ body and provide the incentive to engage in regular PA. It is also important for individuals to get to know their bodies in order to understand when to stop, recognise their own limits and based on their physical reactions, engage in and adapt PA to their own needs. This is how body awareness is developed and alliances between individuals and their bodies created. The ability to slow down and prioritise recovery is as vital as engaging in PA.

I believe I know my body and know what real pain is like. The other pain (soreness from physical activity) is like headache, it does hurt of course, but it is not real (Loppenthin *et al*^34^)

But not overdo it, I think you know when to stop yourself and not to push yourself too hard (Larkin *et al*^16^)

freedom, I can feel myself how my strength is giving out of my body’s saying ‘No’, the physical therapist can’t know this so exactly (Swärdh *et al*^17^)

well just then it was pool gymnastics … it’s at high level… you’re pushed really hard for half an hour. I mean, I know you can also take it easy here, but that’s not what I want because here I can stretch myself to the limit … I get an outlet for this moving about thing, that’s what we’ve got bodies for (Brodin *et al*^14^)

I got to exercise based on what I could manage, you could say. But anyhow, I got… when I had gotten started, the opportunity to try different things, so to say. It wasn’t just for me to do certain things, but I could add or try what worked for me, and that felt good for me. I thought that was good (Lange *et al*^30^)

### Dealing with social support

This theme includes positive and negative aspects of social support related to PA. PA in a clinical environment with support from a physical therapist can be highly valued, since it provides a sense of guidance and supervision, shared responsibility and expertise regarding safety. HPs must also make external demands regarding the adoption and maintenance of PA behaviour in case the internal motivational drives are insufficient. In contrast, support from HPs can be negative in the case of individuals with a high level of autonomy and independence who want to make their own decisions and be responsible for their own regular PA. A sense of not wanting to join regular gyms or exercise groups with healthy people confers a desire to receive social support and be part of exercise groups for people with rheumatic diseases. Such groups also motivate and serve as a discussion forum for the disease itself. However, peer support is not always appreciated due to varying social needs. PA is encouraged through positive attitudes and support from family members, although support will be sought elsewhere in the event that family members are worried and limit the opportunities for PA.

It was so tough as I wanted to do what I know I could do. But I just couldn’t. I would get into fights a lot with (my husband) because of that. I was stressed and sad and the he would be stressed and worried that I had done too much (Baxter *et al*^28^)

Just this help, that you’ve got someone who says, “now you’ve done this,” and you move on…you need encouragement (Swärdh *et al*^17^)

I do not want to go somewhere where it is just about disease and arthritis—for me it is also important to be with ordinary people(Loeppenthin *et al*^34^)

I prefer the group because…it’s quite nice to have other people around you with the same problems, doing the same thing (Withall *et al*^36^)

I mean, we have an email group, so we’ve been emailing each other from time to time with status updates, because several of us had been unwell at the time and we thought it would be a good idea to keep each other updated so you don’t feel left out, or psych each other up to going back… Because we benefit from pushing one another a bit…I’m thinking it keeps us motivated (Swärdh *et al*^33^)

I think that the physiotherapist was very supportive and encouraging, and pushing at the same time. She told me how it should be and what I should try… and it felt safe. That’s how I would describe the physiotherapist. I felt that she had the situation in her hand in some way (Lange *et al*^30^)

### Feeling satisfied with circumstances and achievements

This theme refers to both internal and external aspects of being content with PA arrangements. It is very important to enjoy PA in a pleasant environment with suitable timing, weatherconditions, facilities, as well as convenient travel and economic arrangements according to individual preferences. A sense of independence and autonomy is also essential in order to avoid developing a negative attitude towards PA. A sense of pride and satisfaction with succeeding with regard to the challenges of PA strengthen the individual’s self-efficacy for maintaining such behaviour. A positive mindset towards PA, with a sense of well-being and confidence, will promote long-term commitment to PA.

It has given me confidence, this feeling, well, I still can (Loppenthin *et al*^34^)

the climate is very important (for physical activity), this past summer has been very favourable, 37 degrees (Celsius) out of doors is just right (Kamwendo *et al*^15^)

it builds up your confidence, if you know you are doing the right things for your body, you know what I mean? And then if you see progress, you feel your confidence build up (Bearne *et al*^27^)

I always believe in getting things done early in the morning. I’ve always done that, so it was the best way of doing it (Nichols *et al*^32^)

And i’ll feel better because once I get out and get the air into my head and get my head cleared and you know that’s what it’s all about…It’s absolutely brilliant to get out there and feel the air and listen to the birds (Larkin *et al*^16^)

The question is if I should keep going to [the intervention gym]. I have to go by car since there is a distance. Or should I find another gym? At [the intervention gym] there were space and there were always people, it was clean and tidy, lockers and so, clean and tidy in the showers and it felt great. I have visited another gym and I can’t imagine going there, it felt… no. So that matters, at least to me, how it looks at the gym (Lange *et al*^30^)

## Discussion

Our study is the first to synthesise a wide range of phenomena related *to PA* as perceived by people with RA. Our findings illustrate that the agile adoption and maintenance of PA needs an open and humble attitude towards change, in which the answers are not always available in the moment and in which the process does not always turn out as expected. The disease itself requires flexibility, although not too much, in order to keep up with flares and other unpredictable events. Instead, knowledge, capabilities and insight will gradually develop throughout the process. While in the previous thematic synthesis, which was limited to PA participation, Riggs *et al* 18 described knowledge and guidance from HP as the overarching factor our synthesis illustrates how the constant presence of the disease itself underlies the entire process surrounding of a life with RA, with or without regular PA. Thus, our findings consolidate a previous synthesis of studies with qualitative design that focused on experiences of living with RA.^38^ Symptoms are there described as varying and unpredictable, and the disease as controlling the body, resulting in a life of hardship whereby a person may have to redefine themselves in terms of their illness.^38^

**Figure 3:**
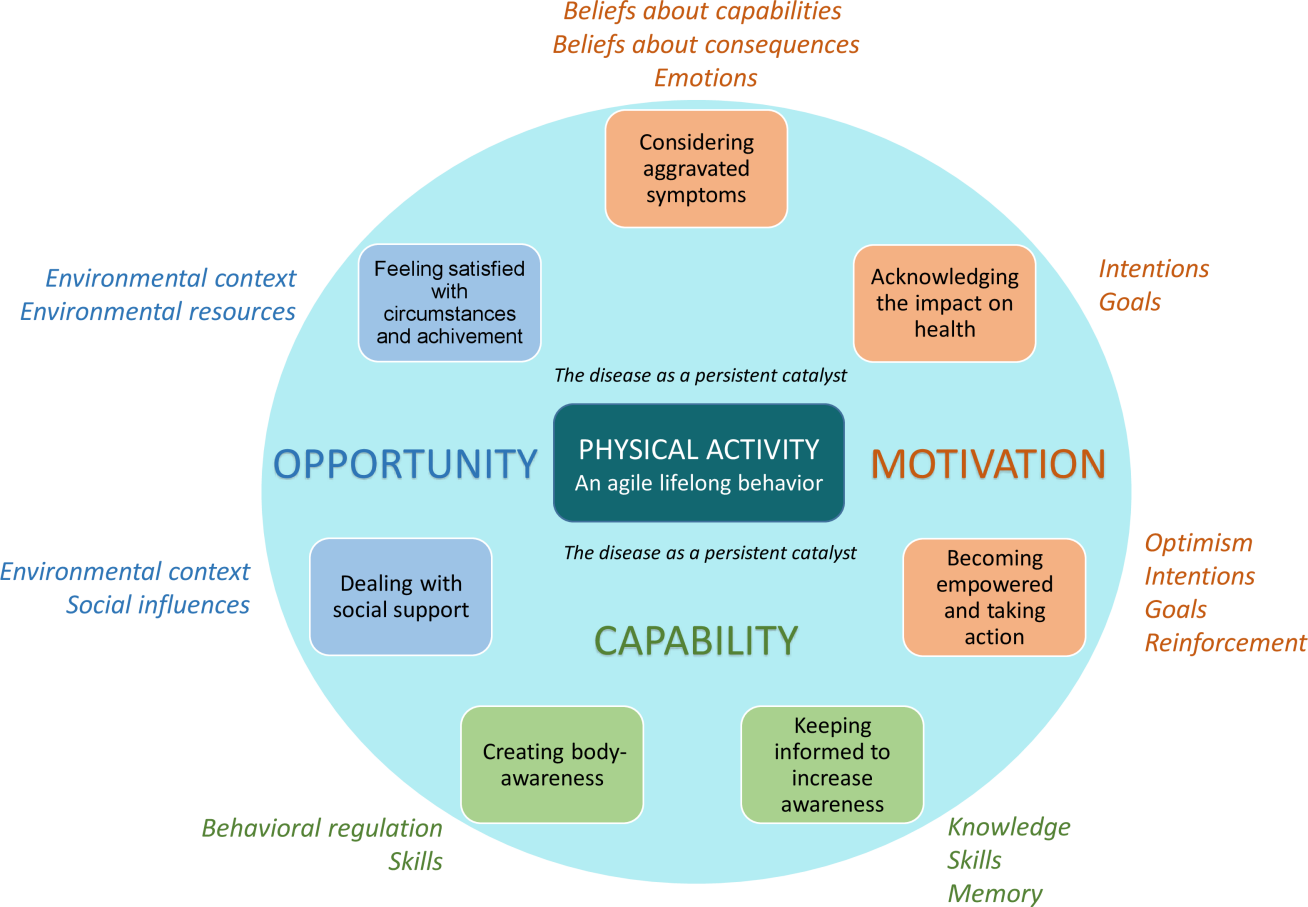
Synthesis of PA perception in RA, interpreted in the light of the behaviour change wheel drivers (capitals) and theoretical domains framework determinants (italics). PA, physical activity; RA, rheumatoid arthritis.

In order to go beyond only summarising and verifying the results, using theory to understand the findings from meta-synthesis investigations could facilitate their implementation into clinical practice.^39^ Our findings clearly illustrate how the three main drivers of behaviour described in the Behaviour Change Wheel (BCW)^40^ are perceived and articulated in the context of PA in RA (figure 3). All our subthemes, interpreted as facilitators and/or challenges for lifelong PA behaviour, describe, resemble and can be explained by the BCW drivers ‘social and physical opportunities’, ‘automatic and reflective motivation’ and ‘physical and psychological capability’ for engaging in PA. Thus, our subthemes can interact in such a way that, in order to engage in regular PA, a patient would have to know more about why it is important (capability), have more access and support to engage in it (opportunity), and/or believe that it would be beneficial and not harmful (motivation). Using the BCW as an explanatory model might thus enhance decisions on the specific drivers to address, based on a certain individuals’ perceptions, for the adoption and maintenance of healthy PA in RA. Furthermore, our subthemes illustrate perceptions of the potential determinants of behaviour, as explained by the Theoretical Domains Framework (TDF)^41 42^ which underpin the BCW drivers (figure 3). These determinants (TDF) can provide guidance on strategies that can be used to modify the behaviour and include individual-level factors.

### Clinical implications

Previous studies on phenomena related to PA in RA identified a range of perceptions on PA, but our synthesis suggests that PA can be understood as an overall agile behaviour with the disease affecting PA by prompting different facilitators and/or challenges. In clinical practice it is important to address them by acquiring structured information on the patients’ previous successes and failures in adopting and/or maintaining a healthy PA behaviour. Linking the patient perceptions of PA to theory (figure 3) and, in a subsequent step, to specific behaviour change techniques, such as education, training, enablement, persuasion, environmental restructuring, modelling, incentivisation or coercion/demands^43^ would provide structured support for both HPs and patients if implemented into clinical practice. It would hence enhance HPs’ understanding of who needs to do what, when, where and how often, to develop facilitators or to overcome perceived challenges related to PA. Thus, some patients may need education while others will benefit from skills training, persuasion or role modelling, for example. Due to the constant presence of the disease, the perceptions described as drivers and determinants of PA behaviour may not always act in the same way or in the same time frame for all patients with RA. However, they will still serve as key elements in conjunction with the disease in the development of this agile lifelong behaviour and will therefore be important to identify when tailoring PA support to a specific patient by selecting appropriate behaviour change techniques.

### Methodological considerations

In order to allow for a wide variety of perceptions of PA, no limits were used in our literature search regarding time or country of origin of the studies. The risk of including older studies from a time when PA was not as accepted by patients with RA as it is today, is probably negligible since the oldest study included was published in 1999. Studies on mind-body PA such as yoga, tai-chi and chi-gong were not included in our synthesis since they are expected to be perceived differently than regular muscle strengthening or cardiorespiratory PA. Studies using e-Health or m-Health technology were excluded since they tend to focus on the content, features, user friendliness and the outcome of the service per se rather than on PA. Thus, our findings are not transferable to these types of PA or PA support. One strength of our synthesis is that we did not exclude any studies because of low CASP grading. However, in order to optimise the quality of the results, we did not include unreviewed grey literature, which might be a limitation. A challenge in the present study was the different epistemological views applied in the original studies that might have generated different kinds of knowledge. However, despite different underlying epistemology, the research methodologies used, for example, data collection and data analysis had more similarities than differences which implies that findings across the studies could be interpreted and synthesised meaningfully.^20^ We also found some heterogeneity in the sampling methods and the samples included in the original studies, which we consider positive since it adds to a broad illumination of the phenomenon under study. Since the participants’ levels of PA were not presented as a variation sampling criterion, a bias towards recruiting participants with similar experiences and/or levels of PA may have occurred in some of the included studies, which might reduce transferability.

### Future research

Many studies that have explored aspects of PA and that span over a period of 19 years were identified in our literature search. Similar themes found across these studies indicate saturation in the area and raise doubts about the need for further replicating research.^44^ Rather, an exploration of the perceptions of innovative interventions based on our synthesis of patient perspectives, linked to theory, could create a basis for reaching beyond current practice. This should also be developed according to global changes that require sustainability by using digital solutions for PA support. The findings of the present synthesis might also be transferable and useful for HPs working with individuals with other inflammatory rheumatic diseases in which PA is also a cornerstone of the treatment. However, in such diseases, other aspects of both drivers and determinants might be present, which would need to be explored in future studies.

## Conclusion

This synthesis has identified several phenomena related to PA in RA and describes PA as an agile lifelong behaviour in which the disease pervades all aspects of an individuals’ perception of PA. Placed in a theoretical context, our findings outline a model for tailoring PA support to the drivers and determinants of a certain individual, which will improve clinical practice for the benefit of both HPs and patients with RA.

## Data Availability

Data are available on reasonable request.
