# Supplementary material for: Physical activity in patients with rheumatoid arthritis - an agile lifelong behaviour: a qualitative meta-synthesis
Source: RMD Open. 2021 May 7;7(2):e001635. doi: 10.1136/rmdopen-2021-001635 (PMC8108693; doi:10.1136/rmdopen-2021-001635)
Supplement: Supplementary data [file rmdopen-2021-001635supp001.pdf]

Supplementary file 1. Checklist for enhancing transparency in reporting the synthesis of qualitative research (ENTREQ)

**No**	**Item**	**Covered = ✓, not covered = x**
**1**	Aim	✓
**2**	Synthesis methodology	✓
**3**	Approach to searching	✓ The search was purposeful.
**4**	Inclusion criteria	✓
**5**	Data sources	✓
**6**	Electronic Search strategy	✓
**7**	Study screening methods	✓
**8**	Study characteristics	✓
**9**	Study selection results	✓ A modified PRISMA flowchart based on purposeful search.
**10**	Rationale for appraisal	✓
**11**	Appraisal items	✓
**12**	Appraisal process	✓
**13**	Appraisal results	✓
**14**	Data extraction	✓
**15**	Software	Not applicable.
**16**	Number of reviewers	✓
**17**	Coding	✓
**18**	Study comparison	✓
**19**	Derivation of themes	✓
**20**	Quotations	✓
**21**	Synthesis output	✓
