# Supplementary material for: Physical activity in patients with rheumatoid arthritis - an agile lifelong behaviour: a qualitative meta-synthesis
Source: RMD Open. 2021 May 7;7(2):e001635. doi: 10.1136/rmdopen-2021-001635 (PMC8108693; doi:10.1136/rmdopen-2021-001635)
Supplement: Supplementary data [file rmdopen-2021-001635supp002.pdf]

Supplementary file 2.

Search terms in Pubmed	arthritis, rheumatoid (MeSH)
	exercise (MeSH), physical activity
	qualitative stud*, interview (MeSH), focus group (MeSH), qualitative method*
Search terms in CINAHL	SH arthritis, rheumatoid
	SH exercise, SH physical activity
	SH qualitative studies, SH interviews, SH focus groups, qualitative methods
Search terms in Web of Science	rheumatoid arthritis
	exercise, physical activity
	qualitative study, interview, focus group, qualitative method

MeSH=medical subject headings (for PubMed), SH= subject headings (for CINAHL). Initial search was performed on March 30, 2019 and additional search was performed on December 31, 2020.
